# LDDP-Net: A Lightweight Neural Network with Dual Decoding Paths for Defect Segmentation of LED Chips

**DOI:** 10.3390/s25020425

**Published:** 2025-01-13

**Authors:** Jie Zhang, Ning Chen, Mengyuan Li, Yifan Zhang, Xinyu Suo, Rong Li, Jian Liu

**Affiliations:** Mechnical and Vehicle Engineering, Hunan University, Changsha 411082, Chinalimengyuan@hnu.edu.cn (M.L.); jyylirong@hnu.edu.cn (R.L.);

**Keywords:** deep learning, defect detection, feature fusion, lightweight network, semantic segmentation, digital images, spatial resolution

## Abstract

Chip defect detection is a crucial aspect of the semiconductor production industry, given its significant impact on chip performance. This paper proposes a lightweight neural network with dual decoding paths for LED chip segmentation, named LDDP-Net. Within the LDDP-Net framework, the receptive field of the MobileNetv3 backbone is modified to mitigate information loss. In addition, dual decoding paths consisting of a coarse decoding path and a fine-grained decoding path in parallel are developed. Specifically, the former employs a straightforward upsampling approach, emphasizing macro information. The latter is more detail-oriented, using multiple pooling and convolution techniques to focus on fine-grained information after deconvolution. Moreover, the integration of intermediate-layer features into the upsampling operation enhances boundary segmentation. Experimental results demonstrate that LDDP-Net achieves an mIoU (mean Intersection over Union) of 90.29% on the chip dataset, with parameter numbers and FLOPs (Floating Point Operations) of 2.98 M and 2.24 G, respectively. Comparative analyses with advanced methods reveal varying degrees of improvement, affirming the effectiveness of the proposed method.

## 1. Introduction

LED chips serve as the foundational components in LED lighting systems and find widespread applications in diverse fields such as lighting, displays, indicators, and signage. Typically crafted from semiconductor materials such as gallium arsenide (GaAs), gallium phosphide (GaP), gallium nitride (GaN), or silicon carbide (SiC), LED chips offer distinct advantages over conventional lighting technologies. One notable advantage lies in their exceptional energy efficiency, as LED chips convert a higher percentage of electrical energy into light compared to incandescent bulbs or fluorescent lamps. Additionally, LED chips exhibit a compact form factor and can be arranged in arrays to generate various light patterns and intensities. This inherent flexibility in design allows for a broad spectrum of lighting solutions. The impact of LED chips on the lighting industry is transformative, introducing unparalleled energy efficiency, durability, and versatility, qualities that traditional lighting technologies struggle to match [1,2,3]. This innovation continues to propel advancements in lighting technology, gaining increased adoption in residential, commercial, and industrial settings worldwide. As the utilization of LED chips continues to rise, the detection of defects becomes paramount during the production process to ensure the overall quality of LED chips [4,5].

In recent decades, significant research attention has been devoted to computer vision processing methods for chip defect detection [6,7,8]. Early studies employed automatic optical inspection (AOI) to identify chip defects. Lin [9] introduced a wavelet-based multivariate statistical approach for detecting ripple defects on chip surfaces. Xie et al. [10] innovatively proposed a golden-template self-generating technique for detecting potential defects in periodic two-dimensional wafer images. This technique involves directly detecting a golden template of the patterned wafer image under examination, requiring no prior knowledge. Zhang et al. [11] presented a hybrid approach for in-tray chip defect inspection, incorporating an image alignment algorithm and a hybrid method for defect detection. Chen et al. [12] developed a microscopic 3D volumetric topographic method based on the infrared confocal principle. In this method, confocal microscopy using infrared (IR) illumination projects active structured light onto the IC sample, enabling 3D volumetric inspection of its internal structure or detected defects. While traditional image processing methods can satisfy the requirements of chip defect detection, they face challenges in meeting the demands of large-scale LED chip detection due to issues such as low efficiency, poor accuracy, and limited robustness.

With the development of deep learning, a series of applications in the field of chip defect detection have been developed by combining the advantages of deep learning, such as stronger learning ability and being data-driven. Nowadays, target detection algorithms are mainly classified into end-to-end-based methods and region-nomination-based methods [13,14]. End-to-end-based methods such as SSD [15] and YOLO [16] obviate the need for region nomination and directly provide the class probability and location coordinates of the target. Huang et al. [17] proposed a novel small-object detection approach utilizing YOLOv4, which aims to enhance detection accuracy. It incorporates expanded feature fusion, optimized anchor box parameters via k-means++ clustering [18], and streamlined YOLO head network branches for improving efficiency. Li et al. [19] proposed an improved chip detection model based on the YOLOv5 network, namely YOLO-STPN. It adds a prediction head to the original model and integrates the swin transformer block [20] and convolutional block attention module into the path aggregation network. The mean average precision at 50% IoU (mAP50) of YOLO-STPN is 10.05% higher than the original YOLOv5, while the decrease of frames per second (FPS) is acceptable. Wang et al. [21] proposed an improved bubble defect detection model, YOLO-Xray, based on the YOLOv5 algorithm for chip X-ray images. The mean average precision (mAP) of the YOLO-Xray algorithm on the CXray dataset reaches 93.5%, which is 5.1% higher than the original YOLOv5. Cao et al. [22] proposed a real-time chip package surface defect detection method based on the YOLOv7 model to solve the challenge of detecting small targets and proposed a confidence propagation cluster (CP-Cluster) to further increase detection accuracy and result confidence. The two-stage classification algorithm has higher detection accuracy, and the single-stage regression algorithm has faster detection speed. Region-nomination-based methods require the preselection of regions of interest and classification detection, such as R-CNN [23], Faster-RCNN [24], and so on. Wang et al. [25] combined the CNN-based super-resolution (SR) technique and a classification model for the application of scanning acoustic microscopy (SAM) to conduct an intelligent inspection of flip chips, which was employed to enhance the SAM image resolution of flip chips, and designed a CNN-based network to classify the solder joints without manual feature extraction. These methodologies often attain elevated levels of precision, but they entail substantial computational expenses, thereby rendering them unsuitable for real-time applications in many cases. While the R-CNN algorithm has significantly enhanced accuracy compared to traditional detection algorithms, its major drawback is evident: redundant computation of overlapping frame features slows down network-wide detection. Despite the Faster R-CNN algorithm’s improved detection accuracy, its reliance on a selective search algorithm to locate regions of interest often results in sluggish performance. Additionally, Faster R-CNN continues to grapple with computational redundancy, and selecting an inappropriate IOU threshold can lead to issues such as noise detection or overfitting. There are also some hybrid algorithms that have been proposed. For example, Zheng et al. [26] proposed a hybrid algorithm based on geometric computation and a convolutional neural network for LED chip defect detection, which improved the SPP network model and performed coarse detection of defects on preprocessed chip lithography graphs in the form of grid segmentation.

Although these target detection algorithms are excellent, they can only determine the bounding box of the defect and cannot quantify the defect. It is important to emphasize that not all defects are categorically prohibited in LED chips. Certain defects must undergo evaluation to ascertain the extent of damage and determine the chip’s qualification based on the nature of the defect, such as surface area loss. Consequently, conventional object detection methods may be constrained in addressing this particular issue.

The primary objective of addressing the defect detection challenge involves establishing a comprehensive framework capable of autonomously analyzing the visual data pertaining to the objects of interest. Due to the irregular nature of defect occurrence, semantic segmentation methods offer a promising alternative. Utilizing the segmentation network, the chip categories are precisely delineated pixel by pixel [27], enabling the detection of damaged areas and evaluation of damage severity. In this way, the quantitative evaluation of defects can be realized. Deep learning has become widely adopted in image processing, with segmentation tasks emerging as a key criterion for evaluating the performance of deep learning models [28,29,30,31]. Shankar et al. [32] introduced a novel defect pattern segmentation scheme designed for enhanced flexibility, leveraging known properties of the human visual system, which remains optimal for image analysis in various applications. Zhang et al. [33] proposed a Non-local Aggregation Network (NANet), with a well-designed Multi-modality Non-local Aggregation Module (MNAM), to better exploit the non-local context of RGB-D features at multi-stage to integrate RGB-D features effectively. Niu et al. [34] proposed a simple plug-and-play data augmentation method based on the requirements of the CNN defect segmentation task to improve the accuracy of the segmentation model. Zhang et al. [35] proposed a lightweight deep learning-based algorithm for chip image segmentation named Hybridformer. It generates scale-aware semantic features using feature maps of different scales as the input part and uses the cross specification and SelfNorm to improve the generalization robustness of the model in terms of distribution bias. The network extracts global contextual information fusing EfficientNetv2 features to achieve accurate localization.

While semantic segmentation methods have demonstrated significant accomplishments in defect detection, our research identifies certain drawbacks. Firstly, in comparison to target detection algorithms, semantic segmentation algorithms exhibit higher parameter counts and slower inference speeds, making it challenging to ensure real-time performance on less powerful computing devices. Secondly, numerous segmentation networks employ 8× or 16× up-sampling directly to restore the feature map to the original resolution, often compromising high-segmentation accuracy. Additionally, during the up-sampling process, the neglect of fine-grained information results in incomplete fusion of feature information. To address these limitations, we propose a lightweight convolutional neural network with dual decoding paths for LED chip segmentation. This innovative approach aims to overcome the aforementioned drawbacks, providing a more efficient and accurate solution for defect detection in the realm of semiconductor chip analysis. The main contributions of this paper can be summarized as follows:

1. A lightweight convolutional neural network with dual decoding paths (LDDP-Net), which achieves good results for LED chip image defect segmentation, is proposed.
2. The backbone of Mobilenetv3 is modified for feature extraction to reduce the number of parameters, and depthwise convolution and pointwise convolution are effectively applied to achieve lightweight.
3. We propose a novel decoder containing dual decoding paths (LDDP), which is comprised of a coarse decoding path and a fine-grained decoding path for parallel operations. Our LDDP can serve as a plug-and-play structure to the efficacy and efficiency of upsampling process.

The remaining sections of the paper are organized as follows. Section 2 describe the details of the proposed method. The strategy of network training, the evaluation metrics, and the experiment results are shown in Section 3. Finally, conclusions are present in Section 4.

## 2. Methodology

### 2.1. Overall Structure of LDDP-Net

**Figure 1:**
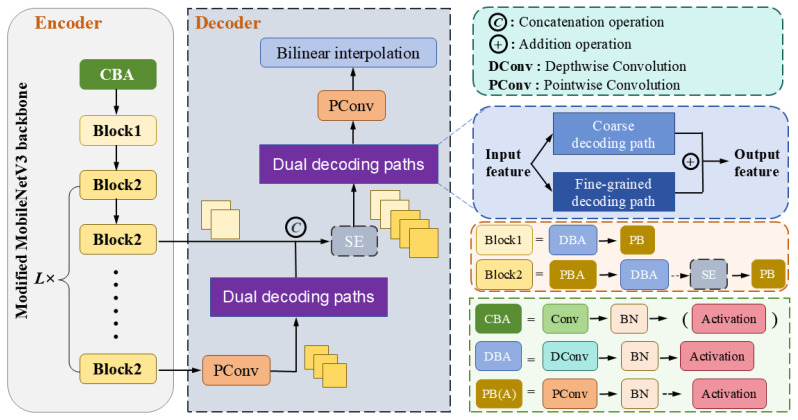
The structure of the proposed LDDP-Net.

Figure 1 illustrates the comprehensive architecture of LDDP-Net, primarily comprised of an encoder and a decoder. The encoder leverages a modified MobileNetV3 backbone, while the decoder is predominantly composed of two intricately designed dual decoding paths to facilitate resolution recovery. Initially, the encoder engages in feature extraction from an input RGB image earmarked for segmentation. During feature extraction, two feature maps are preserved for resolution recovery. The first is the high-layer feature, retaining extensive semantic information for providing global information during up-sampling. The second is the intermediate-layer feature, maintaining intricate details as this location has not undergone complex mapping operations yet. The judicious application of intermediate-layer features serves as a detailed reference for up-sampling. Recognizing that high-level features inherently have a greater number of channels, leading to increased computational load in the decoder, we implement pointwise convolution [36] to reduce the dimension of high-level features. The dimensionally reduced feature map then undergoes the first step of up-sampling through the proposed dual decoding paths. These paths include a simple coarse decoding path, offering macro-information for decoding operations, and a specially designed fine-grained decoding path dedicated to detail reconstruction with the assistance of the coarse path. Subsequently, the up-sampled features are concatenated with the intermediate-layer feature, and the SE block [37] is employed to adjust channel weights. The resulting feature map is fed into the dual decoding paths for the second step of up-sampling. Finally, the required channel number is obtained using pointwise convolution, followed by the bilinear interpolation to ensure the final output aligns with the input.

### 2.2. Modified MobileNetV3 Backbone

**Figure 2:**
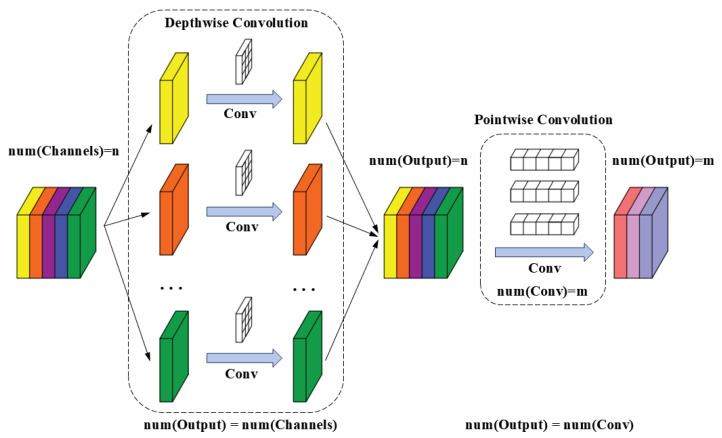
The structure of the depthwise convolution and the pointwise convolution.

Compared to large-scale networks, lightweight networks [38,39,40] are characterized by fewer parameters, reduced computational demands, and shorter inference times, making them more suitable for scenarios with limited storage space and power consumption. Consequently, the study of lightweight networks has garnered widespread attention, with MobileNet [38] serving as a prominent example. MobileNetV3, in particular, stands out for its outstanding performance and speed, showcasing unique advantages across various applications. The lightweight nature of MobileNet can be attributed primarily to depthwise convolution [41] and pointwise convolution [36], whose principles are illustrated in Figure 2. For an input feature map X_in_ with the channel number of C_in_, we can adopt two ways to get an X_out_ with the channel number of C_out_. Let the kernel size be *k*. The number of parameters introduced by conventional convolution can be calculated as

(1) $$C_{in}\times C_{out}\times k\times k$$

However, by decomposing the conventional convolution into a depthwise convolution and a pointwise convolution, the parameter number introduced can be calculated as

(2) $$C_{in}\times k\times k+C_{out}\times C_{in}$$

Furthermore, the detailed structure of MobileNetV3 is determined based on the network architecture search method. However, blindly adopting the model may not yield optimal performance, as the network was originally designed for classification tasks, emphasizing a large receptive field. In the context of segmentation tasks, pursuing an excessively extensive receptive field can be counterproductive, leading to feature information loss. In this study, we have modified the MobileNetV3 backbone to serve as the encoder for chip segmentation, with the detailed structural composition presented in Table 1. Specifically, we set the input image height to 160 pixels and the width to 512 pixels. Within Table 1, the ‘3 × 3’ and ‘5 × 5’ entries in the operator column represent the convolution kernel size for depthwise convolution in this block, respectively. #Out means the number of channels in the middle layer. ‘Exp size’ denotes the number of transition channels from input to output, and the ‘SE’ column indicates whether the SE block is added after depthwise convolution in Block2. In contrast to the original MobileNetV3, we have reduced the downsampling multiple from 32 to 8 to preserve sufficient fine-grained information. Additionally, to reduce network parameters, we have eliminated the last convolution operation in the backbone, resulting in the encoder output channel number being 160 instead of 960.

### 2.3. Dual Decoding Paths

The prevalent upsampling methods primarily fall into the categories of interpolation and deconvolution [42]. Interpolation, devoid of the necessity for introducing additional learnable parameters, stands in contrast to deconvolution, which demands parameter training for determination. The relative performance superiority between these two methods remains ambiguous. Hence, in the formulation of our dual decoding path, we synergistically integrate both approaches.

**Figure 3:**
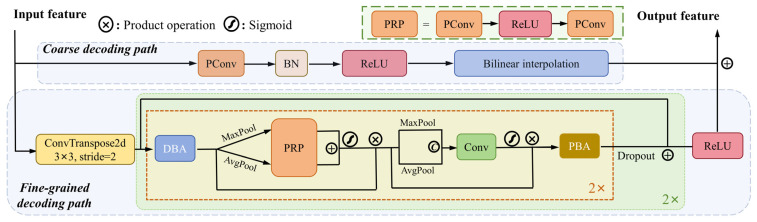
The structure of the proposed dual decoding paths.

As illustrated in detail in Figure 3, the dual decoding paths encompass a coarse decoding path and a fine-grained decoding path operating in parallel. The coarse decoding path simplifies channel reduction and feature map sampling, focusing on furnishing macro-level information, which is crucial for the decoding process. Conversely, the fine-grained decoding path exhibits heightened attention to detail. Employing multiple pooling and convolution techniques, this path focuses the network on spatial locations and channels subsequent to the deconvolution operation. Notably, to curtail the number of parameters and computational load, we exclusively employ depthwise convolution and pointwise convolution within this pathway. This strategic amalgamation of interpolation and deconvolution, combined with the nuanced approaches of coarse and fine-grained decoding, collectively enhances the efficacy and efficiency of our dual decoding framework.

Clearly, the coarse decoding path contains only one convolution combination and one interpolation. For a given input feature X ∈ RC × H × W, the output of the coarse decoding path can be expressed as

(3) $$Y_{c}=Up_{b}(\mathrm{ReLU}(BN(PConv(X))))$$

where *Up*_b_(·) represents the bilinear interpolation. The number of output channels of *PConv* is reduced to 1/2 of the input channels. Therefore, *Y_c_* is the feature map with the channel number, height, and width of 1/2C, 2H, and 2W, respectively.

In the fine-grained decoding path, deconvolution with a stride of 2 effectively reduces the number of channels while doubling the height and width. To enhance the quality of the decoded feature map, we initially employ deconvolution for upsampling. While interpolation optimally utilizes adjacent pixel information, deconvolution results may contain some noise. Consequently, secondary processing of feature maps obtained through deconvolution becomes essential. This secondary processing involves continuous convolution and feature fusion. Let *Y*_1_ ∈ R^1/2C×2H×2W^ be the output after deconvolution. All subsequent operations on *Y*_1_ will not change the output size but will enhance the spatial and channel details. The approach for enhancing the channel details can be expressed as

(4) $$Y_{ch}=f\left[PRP((MP_{ch}(DBA(Y_{1}))))+PRP((AP_{ch}(DBA(Y_{1}))))\right]\cdot DBA(Y_{1})$$

where *MP_ch_* and *AP_ch_* denote the global maxpooling and avgpooling along the channel direction. *Sign*(·) denotes the sigmoid function. The output *Y_ch_* has the same size as *Y*_1_. Then, spatial detail enhancement will be conducted as follows:

(5) $$Y*=PBA(f⎣Conv(CAT(MP_{sp}(Y_{ch})+AP_{sp}(Y_{ch})))⎦\cdot Y_{ch})$$

where *MP_sp_* and *AP_sp_* denote the global maxpooling and avgpooling along the spatial direction. Y^*^ represents the transition feature map. The process is repeated twice and then added to *Y*_1_ after Dropout. The whole process is repeated twice to fully enhance the fine-grained information.

### 2.4. Multi-Layer Feature Fusion

**Figure 4:**
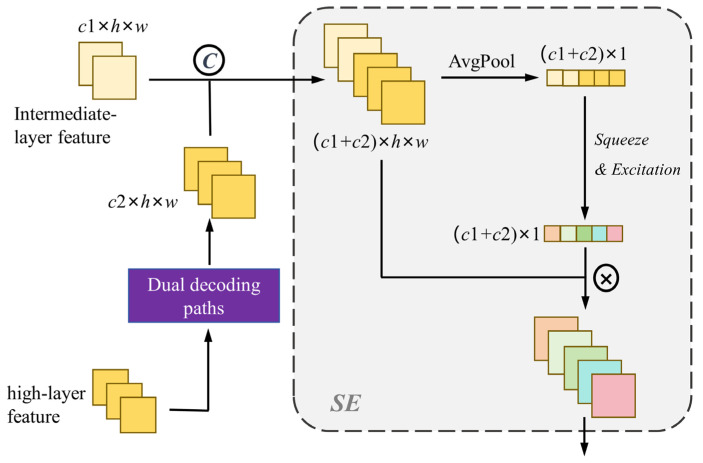
The strategy of the feature fusion.

The high-level feature encapsulates significant semantic information, while the intermediate-level feature is replete with intricate details. Many segmentation networks resort to 8× or 16× direct up-sampling to restore the feature map to its original resolution, posing challenges in achieving high segmentation accuracy. Furthermore, during the up-sampling process, the neglect of details hinders the complete fusion of feature information. For addressing this challenge, our approach involves the initial concatenation of multiple layers of features. Recognizing that simple feature concatenation alone may not effectively adjust their relative importance, we subsequently incorporate the SE block to modulate the weight of individual channels. The schematic representation of the multi-layer feature fusion we employ is depicted in Figure 4.

Given the intermediate-layer feature *X_I_* with the size of *c*1 × *h* × *w* and the high-layer feature up-sampled by dual decoding paths *X_H_* with the size of *c*2 × *h* × *w*, the features concatenated along the channel direction are denoted as *X_cat_*, *X_cat_*∈R^(c1+c2)×h×w^. Next, we perform a self-attention operation on *X*_cat_, which requires squeeze and excitation to get a set of channel weights. The channel weights *w* can be calculated as follows:

(6) $$w=F_{ex}(F_{sq}(AP(X_{cat})))$$

where *AP* denotes global average pooling. *F_sq_*(·) and *F_ex_*(·) denote squeeze and excitation operations, respectively. Both squeeze and excitation operations reduce and increase the data dimension through linear mapping. The channel numbers of *w* are the same as *X_cat_*. Finally, *X_cat_* is multiplied by the weights of the corresponding channels to obtain the result of feature fusion.

## 3. Experimental Results

This section begins by presenting the experiment setup, encompassing implementation details, dataset specifications, and evaluation metrics. Subsequently, the experimental results are comprehensively reported. A detailed analysis of the results is conducted, leveraging both the experimental data and visualizations of the segmentation results from different networks for a qualitative performance comparison.

### 3.1. Experiment Setup

#### 3.1.1. Implementation Details

The camera was a Hikvision MV-CE200-10UC(Haikang in Hangzhou, China), the lens was a 2-magnification video clear, and the WWK20-110C telecentric lens(Vicoimaging in Huizhou, China) had a field of view of 6.4 mm × 4.8 mm. The light source was FG-DVR3W-W (Fugenmv in Shanghai, China) with a white point light and FGTHP100-W(Fugenmv in Shanghai, China) with a white backlight, and the DD motor was a direct-drive motor model DMN71-P0 from Shangyin in Shanghai, China.

LDDP-Net was built under the Pytorch2021.3.3 framework, and all networks were loaded with pretrained weights from ImageNet 1K [43] before training. The SGD [44] with weight decay of 1 × 10^−4^ and momentum of 0.9 was adopted as the optimizer for training. We used the learning rate scheduling *lr* = *baselr* × (1 − *iter*/*total_iter*)*^power^*. The base learning rate was set at 0.025, and the power was set to 0.9. The training epoch on the chip dataset was set to 50 for the networks. The warm-up strategy [45] was used in the first 40 iterations to accelerate the convergence. In addition, the hardware configuration was one single NVIDIA 1650 SUPER GPU (4G Memory). The loss function was dice loss [46]. In order to balance the segmentation difficulty, we set different weight coefficients for different categories. Let *P* be the prediction of the network after softmax and *T* be the true segmentation label. The final loss function formula is as follows:

(7) $$Dice_{i}=\frac{2\left|P_{i}\cap T_{i}\right|}{\left|P_{i}\right|+\left|T_{i}\right|}$$

(8) $$L=\frac{1}{N}\sum_{i=1}^{N}\alpha_{i}(1-Dice_{i})$$

where *i* is the category index, *N* is equal to the total category number, and *α* is the weight coefficient.

#### 3.1.2. Dataset

We collected 150 defective chip images and annotated them using Labelme. Each image was segmented into five categories: background, lead, electrode, light-emitting area, and defect. To ensure uniformity, all chip images were resized to 160 × 512, based on their shape characteristics, before being inputted into the segmentation network. While the shape and position of defects varied significantly, other categories exhibited strong consistency, leading us to forgo additional preprocessing methods and data augmentation strategies. To mitigate potential variations in the segmentation of specific test sets, we relied on the average results obtained through five-fold cross-validation as the benchmark for performance comparison.

#### 3.1.3. Evaluation Metrics

The application of semantic segmentation entails pixel classification within the input chip image, and as a result, the accuracy of segmentation plays a crucial role in subsequent defect detection tasks. To enhance the evaluation of different models, we introduced metrics such as Intersection over Union (IoU), mean Intersection over Union (mIoU), and mean Pixel Accuracy (mPA). IoU quantifies the overlap between the predictions of the segmentation network and the ground truth, while mIoU represents the average IoU value calculated individually for each category. Similarly, mPA gauges the average accuracy of pixel classification, calculated separately for each category. They are defined as follows:

(9) $$IoU_{i}=\frac{p_{ii}}{\sum_{j=0}^{m}p_{ij}+\sum_{j=0}^{m}p_{ji}-p_{ii}},$$

(10) $$mIoU=\frac{1}{m+1}\sum_{i=0}^{m}IoU_{i},$$

(11) $$mPA=\frac{1}{m+1}\sum_{i=0}^{m}\frac{p_{ii}}{\sum_{j=0}^{m}p_{ji}}$$

where *m* represents the category number excluding the background, *p_ij_* denotes that a pixel of category *i* is incorrectly predicted to be category *j*, *p_ji_* denotes that a pixel of category *j* is incorrectly predicted to be category *i*, and *p_ii_* means that a pixel of category *i* is correctly predicted to be category *i*.

### 3.2. Overall Performance

#### 3.2.1. Analysis Based on the Performance Indicators

Table 2 presents a performance comparison of the proposed method against several state-of-the-art models. Notably, LDDPNet exhibits remarkable advantages across all performance indicators. The mIoU reaches 90.29%, surpassing FCN [47] by 10.9%, PSPNet [48] by 12.49%, EncNet [49] by 5.96%, GCNet [50] by 5.99%, DeepLabV3 [51] by 7.98%, and SegNet [52] by 2.77%. Among these, FCN, PSPNet, and DeepLabV3 are conventional segmentation networks employing direct 8× or 4× up-sampling for resolution recovery. This approach sacrifices detailed features, leading to suboptimal segmentation accuracy for challenging categories. For instance, the slender structure of the lead and the intricate nature of the defect make it challenging to precisely define the boundary area, resulting in DeepLabV3 achieving IoU values of only 60.30% and 67.98% for these cases, respectively.

EncNet and GCNet represent two state-of-the-art segmentation models leveraging advanced attention mechanisms, yielding mIoU values of 84.33% and 84.30%, respectively. Remarkably, SegNet, an earlier design, achieves high accuracy through multi-stage upsampling, trailing the proposed LDDP-Net by only 2.77%. In terms of model scale, LDDP-Net boasts a mere 2.98 M trainable parameters, just 1/10th of SegNet, with the fewest FLOPs at 2.24 G. This signifies LDDP-Net’s capability for rapid inference on less powerful computing devices. In conclusion, the proposed network showcases superior overall performance.

#### 3.2.2. Analysis Based on Visualization

**Figure 5:**
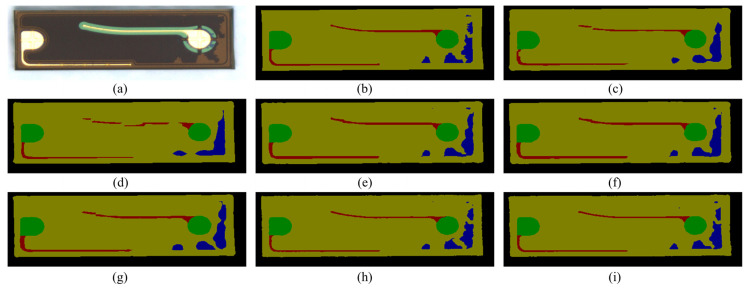
The segmentation result comparison of different models. (**a**) Original image. (**b**) Ground truth. (**c**) FCN. (**d**) PSPNet. (**e**) EncNet. (**f**) GCNet. (**g**) DeepLabV3. (**h**) SegNet. (**i**) LDDP-Net.

The segmentation results obtained using FCN, PSPNet, EncNet, GCNet, DeepLabv3, SegNet, and LDDP-Net are illustrated in Figure 5c–i. It is evident that the segmentation quality aligns with the data distribution provided in the table above for each respective category. Notably, leads and defects prove difficult to distinguish among the five categories. Specifically, leads exhibit a slender appearance, while defects manifest in a variety of shapes and positions. In contrast, the background, light-emitting area, and electrode exhibit relatively fixed positions and shapes.

Observably, FCN, PSPNet, and DeepLabV3 display broken leads, primarily attributable to direct upsampling. GCNet and EncNet incorporate attention mechanisms and context encoding, respectively, resulting in more continuous outcomes. However, lead segmentation remains less accurate due to the width significantly exceeding the ground truth standard.

**Figure 6:**
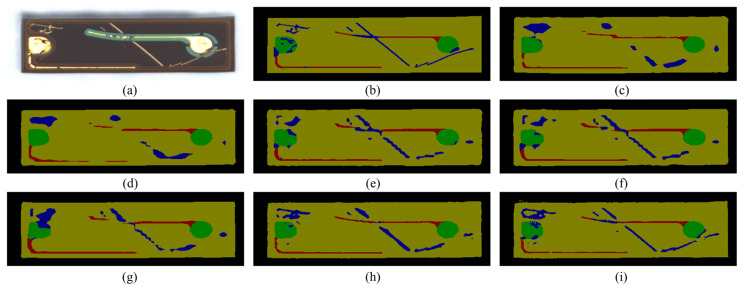
The segmentation result comparison of different models on the complex defect pattern. (**a**) Original image. (**b**) Ground truth. (**c**) FCN. (**d**) PSPNet. (**e**) EncNet. (**f**) GCNet. (**g**) DeepLabV3. (**h**) SegNet. (**i**) LDDP-Net.

Regarding defect segmentation, all methods, except for SegNet and LDDP-Net, struggle with boundary reconstruction. LDDP-Net outperforms SegNet, albeit with some shortcomings. Notably, when confronted with a complex defect pattern, as illustrated in Figure 6, most methods fail to achieve complete segmentation. Despite some limitations in the results obtained by LDDP-Net, it exhibits stronger robustness compared to other advanced methods.

#### 3.2.3. Ablation Experiments

**Figure 7:**
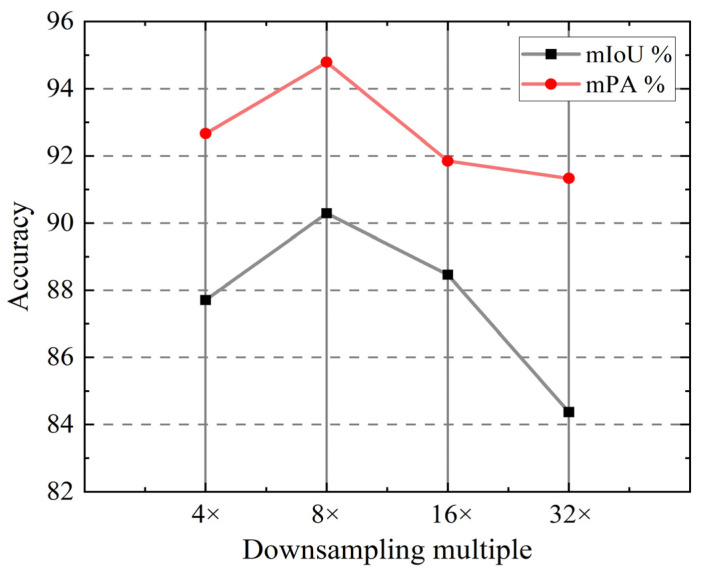
Curves of mIoU and mPA when different downsampling multiples are adopted.

Comparison of Different Downsampling Multiples: In this paper, we introduce a lightweight neural network with dual decoding paths for the segmentation of LED chips. To achieve large receptive fields and preserve low-level semantic information in feature maps, we made modifications to the downsampling multiple of the feature extraction layer. Ablation experiments were performed to identify the optimal network configuration. By controlling the number of strides (stride = 2), various downsampling multiples were obtained. Figure 7 illustrates the curves of mIoU and mPA at 4×, 8×, 16×, and 32×. The original MobileNetV3 utilizes a 32× downsampling, retaining a substantial amount of high-level semantic information crucial for classification tasks. However, in semantic segmentation tasks, the emphasis is on resolution reconstruction. The use of 32× downsampling leads to a noticeable decline in mIoU and mPA. Notably, when the downsampling multiple is set to 8×, the network achieves optimal performance. This configuration strikes a balance between capturing essential high-level features and preserving the necessary details for accurate semantic segmentation.

To evaluate the individual contributions of each pathway in the dual decoding architecture and assess the performance enhancements introduced by the dual decoding design, we conducted ablation experiments focusing on the decoding process. As can be seen in the table, the fine-grained decoding path was significantly higher than the coarse decoding path in the two key indicators of mIoU% and MPa%, which were 4.6% and 1.33% higher, respectively. The IoU of the fine-grained decoding path on the background, lead, electrode, and light-emitting area was also significantly higher than that of the coarse decoding path, especially on the lead, on which the IoU was 21.67% higher. The results demonstrate that the fine decoding path outperforms in most aspects. However, for target regions characterized by a small proportion of features, such as defects, the coarse decoding path exhibits superior performance due to its ability to retain more original features critical for accurate segmentation. As shown in Table 3, the implementation of the dual decoding path effectively integrates the strengths of both decoding paths, resulting in a significant improvement in the overall segmentation performance metrics.

## 4. Conclusions

This paper proposes a lightweight neural network, LDDP-Net, for LED chip segmentation. Within the LDDP-Net framework, modifications to the receptive field of the MobileNetv3 backbone address the challenge of information loss. Additionally, dual decoding paths are introduced, consisting of a coarse decoding path and a fine-grained decoding path. The incorporation of the intermediate-layer feature into the upsampling process significantly enhances the effectiveness of detailed boundary segmentation. Experimental results demonstrate that LDDP-Net boasts the fewest trainable parameters of 2.98M and achieves the highest mIoU and mPA of 90.29% and 94.79%, respectively, on the established chip dataset. The visualization of segmentation results shows that, with the contribution of the dual decoding paths, LDDP-Net can keep the continuity of the segmented target and present the advantage of detail recovery. Although lightweight chip segmentation is realized in this paper, defects are not quantitatively evaluated according to defect types. In future work, further research will be carried out based on the proposed network to realize the deployment and application of the project.

## Figures and Tables

**Table 1:** The detail of structural composition of the modified MobileNetV3.

Input	Operator	Exp Size	#Out	SE	Activation	Stride
160 × 512 × 3	CBA	-	16	-	Hardswish	2
80 × 256 × 16	Block1, 3 × 3	16	16	-	ReLU	1
80 × 256 × 16	Block2, 3 × 3	64	24	-	ReLU	2
40 × 128 × 24	Block2, 3 × 3	72	24	-	ReLU	1
40 × 128 × 24	Block2, 5 × 5	72	40	√	ReLU	2
20 × 64 × 40	Block2, 5 × 5	120	40	√	ReLU	1
20 × 64 × 40	Block2, 5 × 5	120	40	√	ReLU	1
20 × 64 × 40	Block2, 3 × 3	240	80	-	Hardswish	1
20 × 64 × 80	Block2, 3 × 3	200	80	-	Hardswish	1
20 × 64 × 80	Block2, 3 × 3	184	80	-	Hardswish	1
20 × 64 × 80	Block2, 3 × 3	184	80	-	Hardswish	1
20 × 64 × 80	Block2, 3 × 3	480	112	√	Hardswish	1
20 × 64 × 112	Block2, 3 × 3	672	112	√	Hardswish	1
20 × 64 × 112	Block2, 5 × 5	672	160	√	Hardswish	1
20 × 64 × 160	Block2, 5 × 5	960	160	√	Hardswish	1
20 × 64 × 160	Block2, 5 × 5	960	160	√	Hardswish	1

**Table 2:** Comparison of performance indicators of different networks on the chip dataset.

Model	IoU %					mIoU %	mPA %	Params	FLOPs
	Background	Lead	Electrode	Light-Emitting Area	Defect				
FCN [47]	96.21	52.90	93.99	94.30	59.55	79.39	84.93	47.11 M	61.46 G
PSPNet [48]	96.57	48.91	93.80	93.87	55.84	77.80	83.49	46.60 M	55.50 G
EncNet [49]	96.54	62.19	95.23	95.12	72.58	84.33	93.62	33.49 M	43.68 G
GCNet [50]	96.46	61.64	94.81	95.01	73.55	84.30	94.37	47.24 M	61.46 G
DeepLabV3 [51]	97.02	60.30	91.51	94.74	67.98	82.31	87.61	39.64 M	51.23 G
SegNet [52]	97.18	79.59	95.22	96.46	69.18	87.52	92.33	29.44 M	50.35 G
LDDP-Net	97.26	82.30	95.70	97.03	78.75	90.29	94.79	2.98 M	2.24 G

**Table 3:** Comparison of performance metrics for different decoding paths on chip datasets.

Downsampling Model	IoU %					mIoU %	mPA %
	Background	Lead	Electrode	Light-Emitting Area	Defect		
Only coarse decoding path	88.68	56.13	77.79	92.17	61.95	75.34	82.35
Only fine-grained decoding path	96.46	77.81	91.38	94.91	39.15	79.94	83.78
Dual decoding paths	97.26	82.68	95.70	97.03	78.75	90.29	94.79

## Data Availability

Data available on request from the authors.

## References

[1] Shi J., Sun Z. (2016). Large-Scale Three-Dimensional Measurement Based on LED Marker Tracking. Vis. Comput..

[2] Petkovic M., Bajovic D., Vukobratovic D., Machaj J., Brida P., McCutcheon G., Stankovic L., Stankovic V. (2022). Smart Dimmable LED Lighting Systems. Sensors.

[3] Satorres Martínez S., Martínez Gila D.M., Rico S.I., Teba Camacho D. (2023). Machine Vision System for Automatic Adjustment of Optical Components in LED Modules for Automotive Lighting. Sensors.

[4] Shen J., Liu N., Sun H. (2020). Defect Detection of Printed Circuit Board Based on Lightweight Deep Convolution Network. IET Image Process..

[5] Wu C., Zhang W., Jia Q., Liu Y. (2017). Hardware Efficient Multiplier-Less Multi-Level 2D DWT Architecture without off-Chip RAM. IET Image Process..

[6] Jayaram K., Joshi S.S. (2016). Design and Development of a Vision-Based Micro-Assembly System. Proc. Inst. Mech. Eng. Part B J. Eng. Manuf..

[7] Wang J., Zhou X., Wu J. (2021). Chip Appearance Defect Recognition Based on Convolutional Neural Network. Sensors.

[8] Zhou S., Yao S., Shen T., Wang Q. (2024). A Novel End-to-End Deep Learning Framework for Chip Packaging Defect Detection. Sensors.

[9] Lin H.-D. (2007). Computer-Aided Visual Inspection of Surface Defects in Ceramic Capacitor Chips. J. Mater. Process. Technol..

[10] Xie P., Guan S.-U. (2000). A Golden-Template Self-Generating Method for Patterned Wafer Inspection. Mach. Vis. Appl..

[11] Zhang M., Li M., Zhang J., Liu L., Li H. (2020). Onset Detection of Ultrasonic Signals for the Testing of Concrete Foundation Piles by Coupled Continuous Wavelet Transform and Machine Learning Algorithms. Adv. Eng. Inform..

[12] Chen L.-C., Le M.-T., Phuc D.C., Lin S.-T. (2014). In-Situ Volumetric Topography of IC Chips for Defect Detection Using Infrared Confocal Measurement with Active Structured Light. Meas. Sci. Technol..

[13] Zhang D., Wang A., Mo R., Wang D. (2024). End-to-End Acceleration of the YOLO Object Detection Framework on FPGA-Only Devices. Neural Comput. Appl..

[14] Liu X., Lin Y. (2023). YOLO-GW: Quickly and Accurately Detecting Pedestrians in a Foggy Traffic Environment. Sensors.

[15] Kang S.-H., Park J.-S. (2023). Aligned Matching: Improving Small Object Detection in SSD. Sensors.

[16] Hu M., Li Z., Yu J., Wan X., Tan H., Lin Z. (2023). Efficient-Lightweight Yolo: Improving Small Object Detection in Yolo for Aerial Images. Sensors.

[17] Huang H., Tang X., Wen F., Jin X. (2022). Small Object Detection Method with Shallow Feature Fusion Network for Chip Surface Defect Detection. Sci. Rep..

[18] Capó M., Pérez A., Lozano J.A. (2020). An Efficient Split-Merge Re-Start for the K K-Means Algorithm. IEEE Trans. Knowl. Data Eng..

[19] Li K., Xu L., Su L., Gu J., Ji Y., Wang G., Ming X. (2024). X-Ray Detection of Ceramic Packaging Chip Solder Defects Based on Improved YOLOv5. NDT E Int..

[20] Liu Z., Lin Y., Cao Y., Hu H., Wei Y., Zhang Z., Lin S., Guo B. Swin Transformer: Hierarchical Vision Transformer Using Shifted Windows. Proceedings of the IEEE/CVF International Conference On Computer Vision.

[21] Wang J., Lin B., Li G., Zhou Y., Zhong L., Li X., Zhang X. (2023). YOLO-Xray: A Bubble Defect Detection Algorithm for Chip X-Ray Images Based on Improved YOLOv5. Electronics.

[22] Cao Y., Ni Y., Zhou Y., Li H., Huang Z., Yao E. (2023). An Auto Chip Package Surface Defect Detection Based on Deep Learning. IEEE Trans. Instrum. Meas..

[23] Wang S., Xia X., Ye L., Yang B. (2021). Automatic Detection and Classification of Steel Surface Defect Using Deep Convolutional Neural Networks. Metals.

[24] Ren S., He K., Girshick R., Sun J. (2016). Faster R-CNN: Towards Real-Time Object Detection with Region Proposal Networks. IEEE Trans. Pattern Anal. Mach. Intell..

[25] Wang W., Lu X., He Z., Shi T. (2021). Using Convolutional Neural Network for Intelligent SAM Inspection of Flip Chips. Meas. Sci. Technol..

[26] Zheng P., Lou J., Wan X., Luo Q., Li Y., Xie L., Zhu Z. (2023). LED Chip Defect Detection Method Based on a Hybrid Algorithm. Int. J. Intell. Syst..

[27] Li M., Chen N., Suo X., Yin S., Liu J. (2023). An Efficient Defect Detection Method for Nuclear-Fuel Rod Grooves through Weakly Supervised Learning. Measurement.

[28] Ge P., Chen Y., Wang G., Weng G. (2024). An Active Contour Model Based on Jeffreys Divergence and Clustering Technology for Image Segmentation. J. Vis. Commun. Image Represent..

[29] Wang G., Li Z., Weng G., Chen Y. (2024). An Optimized Denoised Bias Correction Model with Local Pre-Fitting Function for Weak Boundary Image Segmentation. Signal Process..

[30] Li M., Chen N., Hu Z., Li R., Yin S., Liu J. (2024). A Global Feature Interaction Network (GFINet) for Image Segmentation of GaN Chips. Adv. Eng. Inform..

[31] Ge P., Chen Y., Wang G., Weng G., Chen H. (2024). A Level Set Approach Using Adaptive Local Pre-Fitting Energy for Image Segmentation with Intensity Non-Uniformity. J. Intell. Fuzzy Syst..

[32] Shankar N., Zhong Z. (2006). A Rule-Based Computing Approach for the Segmentation of Semiconductor Defects. Microelectron. J..

[33] Zhang G., Xue J.-H., Xie P., Yang S., Wang G. (2021). Non-Local Aggregation for RGB-D Semantic Segmentation. IEEE Signal Process. Lett..

[34] Niu S., Peng Y., Li B., Qiu Y., Niu T., Li W. (2024). A Novel Deep Learning Motivated Data Augmentation System Based on Defect Segmentation Requirements. J. Intell. Manuf..

[35] Zhang C., Liu X., Ning X., Bai Y. (2023). Hybridformer: An Efficient and Robust New Hybrid Network for Chip Image Segmentation. Appl. Intell..

[36] Singh P., Mazumder P., Namboodiri V.P. (2022). Context Extraction Module for Deep Convolutional Neural Networks. Pattern Recognit..

[37] Hu J., Shen L., Sun G. Squeeze-and-Excitation Networks. Proceedings of the IEEE Conference on Computer Vision and Pattern Recognition.

[38] Howard A., Sandler M., Chu G., Chen L.-C., Chen B., Tan M., Wang W., Zhu Y., Pang R., Vasudevan V. Searching for Mobilenetv3. Proceedings of the IEEE/CVF International Conference on Computer Vision.

[39] Jia P., Liu F. (2021). Lightweight Feature Enhancement Network for Single-Shot Object Detection. Sensors.

[40] Huang X., Mao Y., Li J., Wu S., Chen X., Lu H. (2023). CRUN: A Super Lightweight and Efficient Network for Single-Image Super Resolution. Appl. Intell..

[41] Gao H., Yang Y., Li C., Gao L., Zhang B. (2020). Multiscale Residual Network with Mixed Depthwise Convolution for Hyperspectral Image Classification. IEEE Trans. Geosci. Remote Sens..

[42] Noh H., Hong S., Han B. Learning Deconvolution Network for Semantic Segmentation. Proceedings of the IEEE International Conference on Computer Vision.

[43] Krizhevsky A., Sutskever I., Hinton G.E. (2017). ImageNet Classification with Deep Convolutional Neural Networks. Commun. ACM.

[44] Yan Z., Chen J., Hu R., Huang T., Chen Y., Wen S. (2020). Training Memristor-Based Multilayer Neuromorphic Networks with SGD, Momentum and Adaptive Learning Rates. Neural Netw..

[45] He K., Zhang X., Ren S., Sun J. Deep Residual Learning for Image Recognition. Proceedings of the IEEE Conference on Computer Vision and Pattern Recognition.

[46] He Z., Pei Z., Li E., Zhou E., Huang Z., Xing Z., Li B. (2024). An Image Segmentation-Based Localization Method for Detecting Weld Seams. Adv. Eng. Softw..

[47] Li G., Liu Q., Zhao S., Qiao W., Ren X. (2020). Automatic Crack Recognition for Concrete Bridges Using a Fully Convolutional Neural Network and Naive Bayes Data Fusion Based on a Visual Detection System. Meas. Sci. Technol..

[48] Sun Y., Zheng W. (2023). HRNet-and PSPNet-Based Multiband Semantic Segmentation of Remote Sensing Images. Neural Comput. Appl..

[49] Zhang H., Dana K., Shi J., Zhang Z., Wang X., Tyagi A., Agrawal A. Context Encoding for Semantic Segmentation. Proceedings of the IEEE Conference on Computer Vision and Pattern Recognition.

[50] Cao Y., Xu J., Lin S., Wei F., Hu H. Gcnet: Non-Local Networks Meet Squeeze-Excitation Networks and Beyond. Proceedings of the IEEE/CVF International Conference on Computer Vision Workshops.

[51] Chen L.-C., Papandreou G., Kokkinos I., Murphy K., Yuille A.L. (2017). Deeplab: Semantic Image Segmentation with Deep Convolutional Nets, Atrous Convolution, and Fully Connected Crfs. IEEE Trans. Pattern Anal. Mach. Intell..

[52] Badrinarayanan V., Kendall A., Cipolla R. (2017). Segnet: A Deep Convolutional Encoder-Decoder Architecture for Image Segmentation. IEEE Trans. Pattern Anal. Mach. Intell..

